# Validation of a Novel EUS-FNB-Derived Organoid Co-Culture System for Drug Screening in Patients with Pancreatic Cancer

**DOI:** 10.3390/cancers15143677

**Published:** 2023-07-19

**Authors:** Simon Ezban Grützmeier, Bojan Kovacevic, Peter Vilmann, Charlotte Vestrup Rift, Linea Cecilie Melchior, Morten Orebo Holmström, Lene Brink, Hazem Hassan, John Gásdal Karstensen, Hanne Grossjohann, Deepthi Chiranth, Anders Toxværd, Carsten Palnæs Hansen, Estrid Høgdall, Jane Preuss Hasselby, Pia Klausen

**Affiliations:** 1 Gastro Unit, Endoscopic Division, Copenhagen University Hospital Herlev and Gentofte, 2730 Herlev, Denmark; bojan.kovacevic@regionh.dk (B.K.); peter.vilmann@regionh.dk (P.V.); lene.brink@regionh.dk (L.B.); hazem.hassan.al-hashemi@regionh.dk (H.H.); pia.helene.klausen.01@regionh.dk (P.K.); 2 Department of Surgery and Transplantation, Copenhagen University Hospital Rigshospitalet, 2100 Copenhagen, Denmark; hanne.grossjohann@regionh.dk (H.G.); carsten.palnaes.hansen@regionh.dk (C.P.H.); 3 Department of Clinical Medicine, University of Copenhagen, 2200 Copenhagen, Denmark; john.gasdal.karstensen@regionh.dk; 4 Department of Pathology, Copenhagen University Hospital Rigshospitalet, 2100 Copenhagen, Denmark; charlotte.vestrup.rift@regionh.dk (C.V.R.); linea.cecilie.melchior@regionh.dk (L.C.M.); deepthi.chiranth@regionh.dk (D.C.); jane.preuss.hasselby@regionh.dk (J.P.H.); 5 National Center for Cancer Immune Therapy, Department of Oncology, Copenhagen University Hospital Herlev and Gentofte, 2730 Herlev, Denmark; morten.orebo.holmstroem@regionh.dk; 6 Department of Immunology and Microbiology, University of Copenhagen, 2200 Copenhagen, Denmark; 7 Pancreatitis Centre East, Gastroenterology Unit, Copenhagen University Hospital—Amager and Hvidovre, 2650 Hvidovre, Denmark; 8 Department of Pathology, Copenhagen University Hospital Herlev and Gentofte, 2730 Herlev, Denmark; anders.toxvaerd.01@regionh.dk (A.T.); estrid.hoegdall@regionh.dk (E.H.)

**Keywords:** pancreatic cancer, patient-derived organoids, EUS-FNB, co-cultures, cancer-associated fibroblasts, personalized medicine, disease modelling

## Abstract

**Simple Summary:**

Pancreatic cancer is a devastating disease with a 5-year survival rate of 12%. Only about 20% of patients are candidates for curative surgery, while the majority receive palliative chemotherapy or best supportive care. Recently, testing chemotherapeutical agents on patient-derived 3-dimensional cultures (organoids) of cancer cells has provided hope that personalized treatment may soon become a reality. However, most of these models only include tumor cells and not cells of the supporting tissue, which have been shown to play a critical role in cancer progression. In this study, we created a co-culture including both tumor and stromal cells from endoscopic ultrasound-guided biopsies and showed that an interaction occurs between the cell types in our model. It may therefore be a step towards better prediction of therapeutic response in the future. We also discuss the limitations of creating these types of models by using endoscopic biopsies from primary tumors.

**Abstract:**

Cancer-associated fibroblasts (CAFs) have been shown to impact the chemosensitivity of patient-derived tumor organoids (PDTOs). However, the published literature comparing PDTO response to clinical outcome does not include CAFs in the models. Here, a co-culture model was created using PDTOs and CAFs derived from endoscopic ultrasound-guided fine-needle biopsies (EUS-FNBs) for potential use in drug screening applications. Co-cultures were established, and growth was compared to monocultures using image metrics and a commercially available assay. We were able to establish and expand validated malignant PDTOs from 19.2% of adenocarcinomas from EUS-FNBs. CAFs could be established from 25% of the samples. The viability of PDTOs in the mixed cell co-culture could be isolated using image metrics. The addition of CAFs promoted PDTO growth in half of the established co-cultures. These results show that co-cultures can be established from tiny amounts of tissue provided by EUS-FNB. An increased growth of PDTOs was shown in co-cultures, suggesting that the present setup successfully models CAF–PDTO interaction. Furthermore, we demonstrated that standard validation techniques may be insufficient to detect contamination with normal cells in PDTO cultures established from primary tumor core biopsies.

## 1. Introduction

Pancreatic ductal adenocarcinoma (PDAC) has a poor prognosis with a 5-year survival of 12% [1]. Only about 15–20% of patients are candidates for curative surgery at the time of diagnosis, leaving chemotherapy as the only treatment option for the majority of patients. Median survival time for patients receiving palliative oncological treatment is only 6–11 [2,3,4] months. In recent years, the prospect of using patient-derived tumor organoids (PDTOs) established from biopsies to guide oncological treatment has gained significant attention. PDTOs derived from EUS-FNBs are of special interest, as tissue is obtained prior to initiation of treatment, and this may allow for optimal choice of first-line therapy. Previous studies have shown that the response of PDTOs to chemotherapeutical agents correlates with clinical response [5,6,7] and that PDTOs can be established from EUS-FNBs [8,9,10,11,12], indicating that the technology has potential for a personalized approach in the treatment of pancreatic cancer. However, standard PDTO models only consist of organoids derived from tumor cells and do not adequately model the tumor microenvironment (TME), of which cancer-associated fibroblasts (CAFs) are a dominant component. It has been widely accepted that the TME—and CAFs in particular—have prominent effects on functional properties such as tumor growth, metastatic potential, and therapeutic response [13,14]. A characteristic feature of CAFs is their ability to produce and remodel extracellular matrix, making CAF–tumor interaction particularly relevant in PDAC, where up to 90% of tumors consist of desmoplastic tissue [15]. Several studies have shown increased therapeutic resistance in PDAC under the influence of CAFs in vitro [16,17,18,19], suggesting that improved modelling of the tumor stroma might have an impact on the predictive capabilities of organoid cultures. To address these shortcomings of current predictive PDTO models, the aim of this study was to develop a next-generation organoid co-culture system derived from EUS-FNBs, combining the advantage of establishing cultures prior to initiation of first-line treatment with improved stromal modelling using CAFs. Herein, we describe methods of isolation of PDTOs and CAFs from EUS-FNB samples, cultivation, validation, and estimation of PDTO viability in a co-culture setting along with the expected success rates and limitations of the model.

## 2. Materials and Methods

### 2.1. Patients and Study Protocol

The study was approved by the Regional Ethics Committee (H-19040233) at The Capital Region, Denmark, and was conducted in accordance with the Helsinki Declaration. All patients provided written informed consent prior to acquisition of tissue. Between October 2020 and December 2022, patients admitted for EUS-FNB procedures of pancreatic lesions were assessed for eligibility. Inclusion criteria were (1) patient age of 18 years or above and (2) imaging findings suspicious of PDAC requiring additional follow-up with EUS-FNB. This included patients with inoperable PDAC who required histopathologic confirmation prior to initiation of chemotherapy as well as patients with atypical solid lesions of the pancreas requiring histological evaluation. Exclusion criteria were (1) inability to obtain informed consent and (2) patients with absolute contraindications to EUS-FNB.

### 2.2. Endoscopic Ultrasound-Guided Fine-Needle Biopsies

EUS-FNB procedures were performed by experienced endoscopists at three expert centers. All biopsies were acquired using 22G FNB needles (SharkCore^®^ (MedTronic, Minneapolis, MN, USA), TopGain^®^ (Medi-globe GmbH, Grassau, Germany), and Acquire^®^ (Boston Scientific, Marlborough, MA, USA)), except for one biopsy where tumor outgrowth was observed at the papilla and where tissue was acquired using a conventional endoscopic biopsy forceps. The slow-pull technique was used in all instances but was supplemented with negative-pressure suction at the endoscopist’s discretion in one case. The fanning technique was used in all instances. Two to three biopsies were acquired for diagnostic purposes, after which an additional 1–2 biopsies were used for PDTO and CAF culture establishment, not exceeding five needle passes in total to limit the risk of bleeding. Biopsies for research purposes were transferred to a collecting medium consisting of AdDF+++ (97% Advanced DMEM/F-12 (12634010, ThermoFisher, Waltham, MA, USA), 1% 200 mM GlutaMAX (35050061, ThermoFisher), 1% 1M HEPES (15630056, ThermoFisher), and 1% 10,000 Unit/mL penicillin/streptomycin) supplemented with 1× Primocin (ANT-PM-1, InvivoGen, Toulouse, France) and transported to our laboratory.

### 2.3. Establishment of PDTOs and CAF Cultures

PDTO cultures were established as described by Driehuis et al. [20] with modifications. Briefly, tumor specimens were incubated in a tissue digestion medium containing AdDF+++ supplemented with 1–5 mg/mL collagenase II (17101015, ThermoFisher) and 10μM Y-27632 (S1049, Selleck Chemicals GmbH, Planegg, Germany) for 5–30 min depending on biopsy composition and intermittently disturbed using mechanical shearing applied by aspiration in a 25G syringe. This was repeated until the larger core biopsy was broken down into smaller tissue fragments. Digestion to single cells was avoided to preserve tumor cells and CAF viability. Use of red blood cell lysis buffer (ab204733, Abcam, Cambridge, UK) was applied if needed. Cells were resuspended in growth-factor-reduced Matrigel^®^ (356231, Corning, NY, USA) or Geltrex^®^ (A1413202, ThermoFisher) and seeded in 7.5 μL domes in 24-well plates with 4 domes/well. (Matrigel^®^ was initially used, but due to global shortage during the COVID pandemic, it was replaced with Geltrex^®^.) After 30 min at 37 °C, the domes were overlaid with 0.5 mL of tumor selective medium (TSM). TSM was based on basal organoid medium containing AdDF+++, 1× B27 supplement, 1 mM N-acetylcysteine (A9165, Sigma-Aldrich, Merck KGaA, Darmstadt, Germany), 10% R-Spondin1 (RSPO1)-conditioned medium, 100 ng/mL Noggin (120-10C, ThermoFisher), 100 ng/mL FGF-10 (100-26, ThermoFisher), and 0.5× Primocin and supplemented with either 500 nM A83-01 (2939, Tocris, Bio-Techne, Minneapolis, MN, USA) and 50% WNT3a-conditioned medium (TSM-WNT^+^) or 50% AdDF+++ and recombinant hEGF (AF-100-15, ThermoFisher) (TSM-EGF^+^). Once established, organoids were passaged as appropriate by dissociation using TrypLE Express (12604013, ThermoFisher) for 2–5 min at 37 °C or mechanical sheer using a syringe.

As the exact diagnosis was unknown prior to patient inclusion, samples from non-pancreatic malignancies (papillary adenoma, neuroendocrine tumors, pancreatitis, metastasis from distant primary tumor, and inconclusive results) were obtained and organoid cultures initiated. In those cases, cultures were discontinued upon receiving histopathological diagnosis. Success rates regarding organoid and CAF cultures were calculated per biopsy diagnostic for primary adenocarcinoma.

For detailed information regarding drug screening experiments, immunofluorescent staining, immunohistochemistry, DNA isolation, next-generation sequencing (NGS), and CAF phenotyping by fluorescence activated cell sorting, see Section S1 in Supplementary Materials.

### 2.4. Isolation of CAFs

CAFs were isolated from the initial passages of PDTO cultures. CAFs were identified microscopically by their spindle-like appearance, and whenever outgrowth was observed, the dome was left in the well when PDTOs were passaged to allow CAFs to expand and attach to the bottom of the plate. After CAF expansion to approximately 80% confluence of the well, the medium was aspirated, and 300 µL Trypsin/EDTA Solution A (0.25%/0.02%) (03-050-1A, Biological Industries, Beit Haemek, Israel) was added to the well for 1–3 min under close observation under the microscope. CAFs would detach from the plastic surface sooner than the tumor cells, allowing them to be separated and collected in the aspirate.

Once CAFs had been isolated, they were cultured in T75 plastic flasks in 89% RPMI 1640 (61870010, Gibco, ThermoFisher), 10% Fetal Bovine Serum (FBS) (A4766801, ThermoFisher), and 1% penicillin/streptomycin 10,000 U/mL (15140122, ThermoFisher) and passaged once 80% confluent.

### 2.5. Co-Culture of PDTOs and CAFs

Once PDTOs had been expanded to a sufficient amount of biomaterial, they were digested into single cells using TrypLE Express supplemented with 10 μM Y-27632 to reduce apoptosis and strained through a 70 µm filter to remove larger cell aggregates. Tumor cells and CAFs were mixed in a 2:1 ratio. Cells were resuspended in TSM, supplemented with 10% Geltrex^®^ and 10 μM Y-27632, in a density of 50 tumor cells/µL. The suspension was seeded on 384-well plates, 20 µL/well. The viability of co-cultures was evaluated after 6 days of growth using CellTiter-Glo^®^ 3D (G9681, Promega, Madison, WI, USA) and by obtaining microscopic images using the Incucyte^®^ S3 Live-Cell Analysis Instrument (Satorius, Göttingen, Germany).

### 2.6. Image Metrics Measurements

Pictures obtained during co-culture and drug screening experiments were evaluated using CellProfiler software version 4.2.1 (Broad Institute of MIT and Harvard, Cambridge, MA, USA) [21,22]. Individual PDTOs were marked manually by reviewers (S.E.G. and P.K.). Reviewers were trained on image sets of monocultures of both CAFs and PDTOs, including positive control wells subjected to staurosporin, to ensure uniform classification of organoids and CAFs. As dead cells could not readily be distinguished from live single cells on images, cultures with poor overall viability (average of <1000 luminescent units/well, as measured by CellTiter-Glo^®^) were excluded. Similarly, detached CAFs were often larger than their tumor cell counterparts and harder to distinguish from small PDTOs. To avoid misclassification, a size limit was selected based on measurement of CAFs from 150 different wells in monocultures of CAFs, and that limit that excluded 95% of CAFs.

### 2.7. Statistical Analysis

All statistical analyses were performed using R version 4.1.1 (R Foundation for Statistical Computing, Vienna, Austria) and Rstudio version 2023.03.0 + 38626 (Posit, Boston, MA, USA). Significance levels were set at 5% for all calculations. Numerical outcomes were compared by *t*-test or Wilcoxon rank-sum test, as appropriate. Outlier measurements were excluded following significance testing using Dixon’s Q-test.

## 3. Results

### 3.1. Establishment of CAF and PDTO Cultures from EUS-FNB Samples

EUS-FNB samples were obtained from three different endoscopic centers, including one in-house endoscopic unit in immediate relation to our laboratory. Transport time for biopsies acquired in-house was 5–10 min and 35–90 min when arriving from the other two centers. The success of PDTO cultures was evaluated by their initial establishment rate (initial outgrowth of organoids), expansion rate (expansion to dense organoid growth in 6–8 wells), and rate of validation (presence pathogenic mutations confirmed by NGS analysis). We found no correlation between decreased cellular viability and increased transport time (up to 90 min), with establishment rates being similar between biopsies arriving immediately and those undergoing brief transportation (establishment rate 90.4% and 100%, respectively; *p* = not significant). Biopsies transported from other centers were initially kept on ice during transport, but later attempts with transport at ambient temperature impacted neither establishment nor expansion rate. Likewise, we did not observe any significant difference between using either one or two needle passes for tissue acquisition in either establishment rate (91.7% and 83.3%; *p* = not significant) or expansion rates (50% both groups; *p* = not significant). Most samples (78%) were acquired using 22G TopGain EUS-FNB needles, and the study was underpowered to detect differences in success rate between needle types.

Contamination with normal epithelial cells was a recurrent issue. During initial attempts at establishing PDTO, complete organoid medium (basal organoid medium supplemented with both WNT3a-CM, A83-01, and hEGF) was used. However, NGS analysis failed to confirm presence of pathological mutations in any of these cultures. Subsequently, we implemented use of TSM-EGF+ and TSM-WNT+, i.e., media with no addition of either WNT/A83-01 or EGF, respectively, for selective malignant cell growth, as described by Driehuis et al. [20,22]. Exclusion of certain growth factors had previously been shown to reduce expansion of normal pancreatic organoids [23,24]. After implementation of TSM, we observed an establishment rate of 92.8%, expansion rate of 50%, and malignant validation rate of 19.2%.

**Figure 1:**
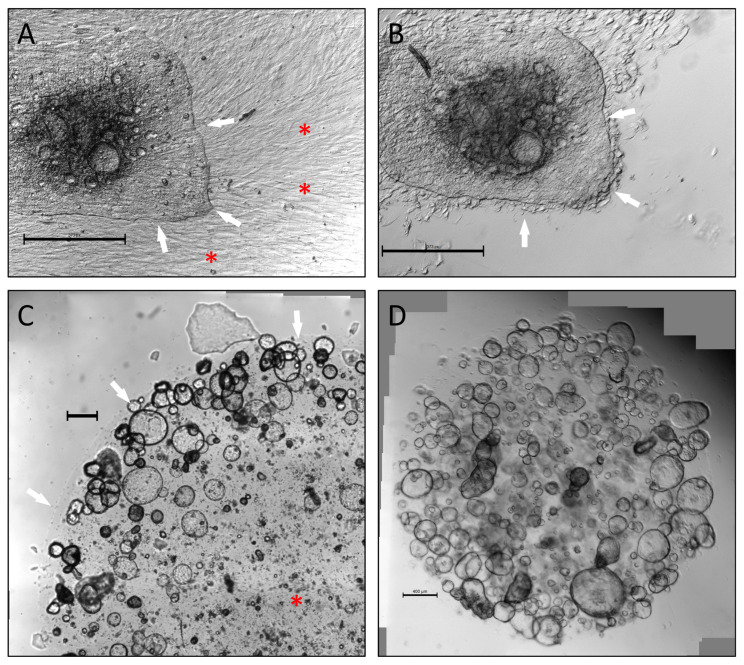
Process of isolating CAFs from epithelial cells. Scale bars 400 µm (**A**) Epithelial border marked by white arrows. The bottom is covered in confluent CAFs (red asterisk). (**B**) Same area following trypsin treatment. Epithelial cells remain attached, while most CAFs have detached.(**C**) Increased growth of organoids observed along edge of 50 µL Geltrex^®^ dome (white arrows); lack of organoid growth centrally (red asterisk). (**D**) The 7.5 µL dome with uniform growth of organoids throughout. CAFs, cancer-associated fibroblasts. Process of isolating CAFs from epithelial cells. Scale bars 400 µm (**A**) Epithelial border marked by white arrows. The bottom is covered in confluent CAFs (red asterisk). (**B**) Same area following trypsin treatment. Epithelial cells remain attached, while most CAFs have detached.(**C**) Increased growth of organoids observed along edge of 50 µL Geltrex^®^ dome (white arrows); lack of organoid growth centrally (red asterisk). (**D**) The 7.5 µL dome with uniform growth of organoids throughout. CAFs, cancer-associated fibroblasts.

To create patient-matched PDTO and CAF cultures from the limited amount of tissue provided by a single EUS-FNB sample, we initially attempted to divide biopsies into two fractions, establishing CAF cultures using the “outgrowth method” [21] from one and another for PDTO cultures. These attempts were unsuccessful due to low cellularity in each fraction. Instead, a higher success rate was achieved by expanding CAFs directly from PDTO cultures. During this process, Geltrex^®^ domes with CAF outgrowth were left undisturbed until CAFs became confluent. CAFs were then left isolated from PDTOs using careful trypsin treatment (Figure 1A,B). In our experience, avoiding complete digestion of biopsies with seeding of small tissue fragments in Geltrex^®^ was most beneficial in terms of achieving CAF outgrowth. Similarly, using small Geltrex^®^ domes (7.5 µL) reduced loss of PDTO material whenever a dome was selected for CAF expansion. Furthermore, use of smaller domes circumvented an issue with central lack of cellular expansion, which was sometimes observed in larger volumes of Geltrex^®^ (50 µL) (Figure 1C,D). Using this method, CAFs cultures could be established from 25.6% of malignant biopsies, with success defined as expanding cells to 80% confluence in a T75 flask. These cultures could be maintained for approximately six to eight passages before becoming quiescent.

### 3.2. Validation and Classification of PDTOs

**Figure 2:**
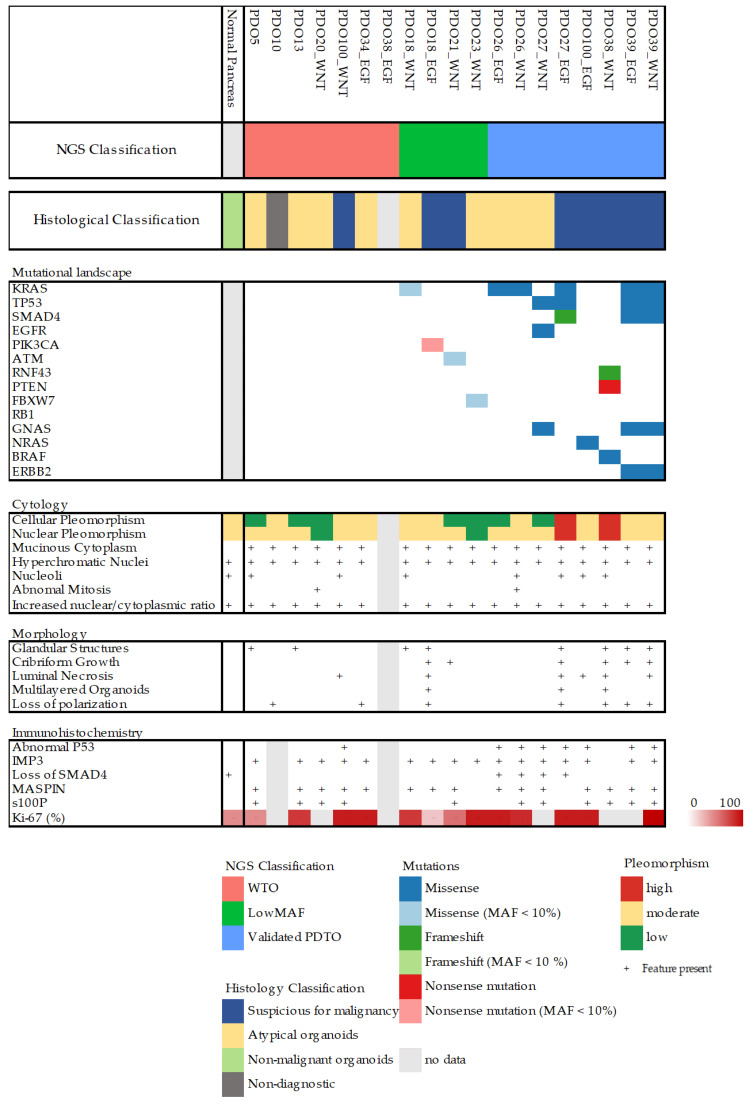
Overview of mutational landscape and histological features of each culture.

All organoid cultures that were successfully expanded were further characterized by an NGS panel (Section S1.5 in Supplementary Materials) and histopathological evaluation. Organoids were classified according to their mutational profile as well as by histopathological evaluation (Figure 2 and Table S1). Organoids without any detectable mutations were classified as wild-type organoids (WTOs), and organoids with pathologic mutations and mutant allele frequency (MAF) < 30% were classified as low-MAF organoids, whereas only organoids with MAF > 30% were classified as validated PDTOs (vPDTOs).

**Figure 3:**
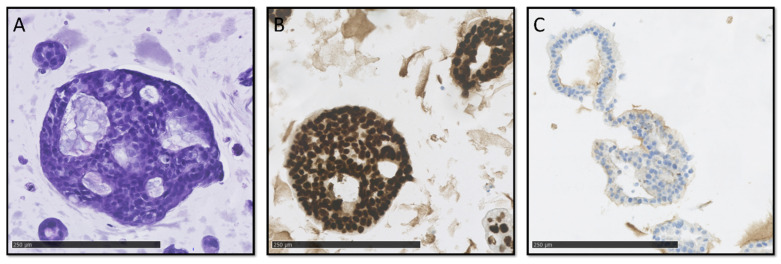
HE and IHC slides. Scale bar 250 µm. (**A**) HE slide of organoids showing cribriform growth. (**B**) Overexpression of P53. (**C**) Complete loss of SMAD4 expression.

Digitized slides were evaluated by trained pathologists (C.V.R., A.T., J.P.H., and D.C.) for several different cytological and architectural malignant characteristics (Figure 2 and Figure 3A–C). There was limited concordance between histopathological and NGS classification. While high-grade nuclear and cellular pleomorphism was only observed in the organoids of the vPDTO group, organoids with both low and moderate pleomorphism could also be observed in the vPDTO group. Similarly, cribriform growth was specific to organoids in low-MAF and vPDTO groups but only present in half of the cultures (Figure 3A). One organoid culture derived from a normal pancreatic surgical specimen was used as a reference. While this culture was the only one that was histopathologically classified as non-malignant, it did also exhibit cellular characteristics usually consistent with malignancy, such as increased nuclear-cytoplasmatic ratio and hyperchromatic nuclei.

Histopathological evaluation of HE-stained FFPE slides was supplemented by a panel of IHC stains. Abnormal p53 expression was observed in all vPDTO cultures (Figure 2, Figure 3B) but also in one culture in the WTO group, which was histologically classified as malignant. We found no combination of histological markers that separated organoids in groups according to NGS classification, demonstrating the difficulties of using histology alone as a validation tool of malignancy.

To assess whether the NGS classification system was valid, a WTO culture that exhibited prolonged growth and expansion in TSM for more than five passages was chosen for supplementary copy-number-variation analysis (Figure S1), but this provided no indication of chromosomal instability, supporting that the culture was classified correctly.

### 3.3. Alternative Methods of Validation

**Figure 4:**
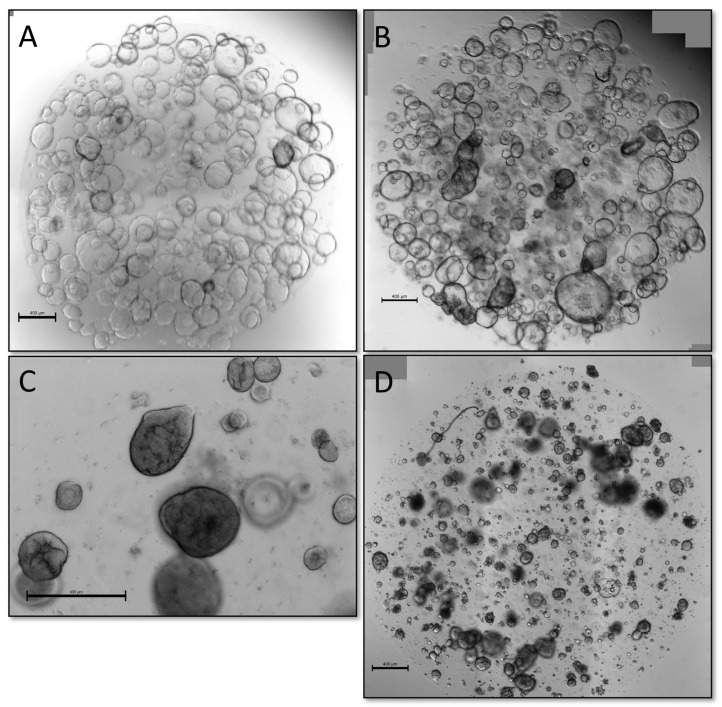
(**A**) WTO culture with cystic morphology, passage 5. (**B**) Validated PDTO primarily with cystic morphology, passage 5. (**C**) WTO culture with solid morphology, passage 2. (**D**) Validated PDTO with cystic morphology, passage 2. WTO, wild-type organoid; PDTO, patient-derived tumor organoid. Scale bar 400 µm.

We also explored alternative methods of validation, namely if organoids could be classified as malignant or benign either by microscopic appearance of live organoids or presence of sustained growth in TSM, defined as the presence of viable organoids for five passages, a previously applied criterium for a “successful culture” in earlier publications [7,8,25]. While WTO cultures often had a cystic appearance with clear, thin-walled organoids (Figure 4A), they could also appear as solid, dense-looking organoids (Figure 4C). Likewise, validated PDTOs could have both cystic (Figure 4B) and solid appearance (Figure 4D), making certain visual distinction between benign and malignant organoids difficult.

**Figure 5:**
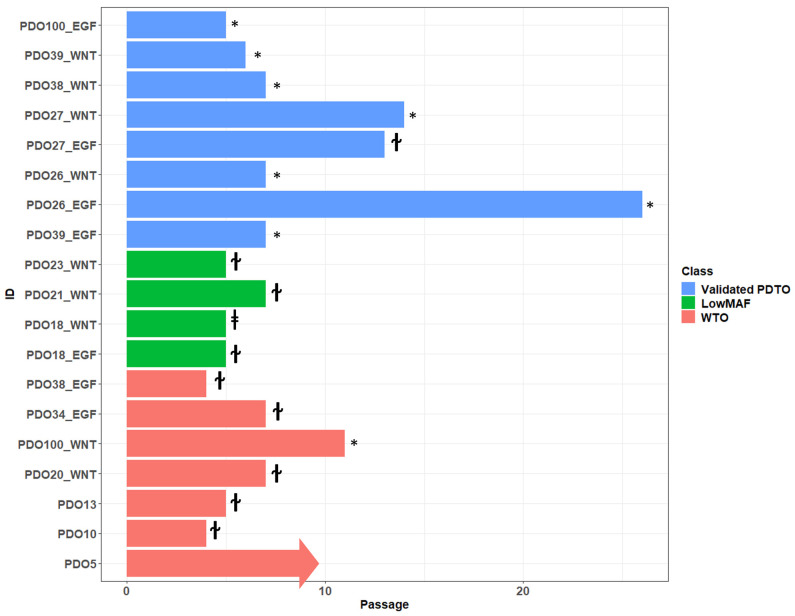
Overview of lifespan of cultures characterized by NGS. Cultures PDO5-PDO13 were initiated in complete organoid medium. ɫ Cessation of growth; * cryopreservation viable state; ^ⱡ^ culture lost due to technical error.

Overview of passages that could be sufficiently expanded for NGS analysis are shown in Figure 5. If cultures were classified as low-MAF or WTO, expansion past passage 5 was attempted to see how long growth could be maintained. In general, these cultures exhibit decreased proliferation at around passage 4 until eventually stopping completely at passage 5–7, although growth as far as passage 10 was observed in one instance before cryopreservation. In total, 9/11 (82%) cultures classified as low-MAF or WTO could be maintained until at least passage 5. The same pattern of initial organoid formation and proliferation, followed by slow decline over several passages, was observed in many cultures that never met our expansion criteria, suggesting that failure of expansion was caused by lack of malignant cells.

Cultures classified as vPDTOs were cryopreserved after downstream analysis and co-culture experiments. In general, vPDTOs showed stable growth at time of cryopreservation, without signs of decreased proliferation. Three cultures were thawed and used for auxiliary experiments, with two of them continuing proliferation for 14+ passages until repeated cryopreservation, with the last culture eventually being terminated due to cessation of growth after 13 passages.

This showed that while malignant PDTOs have increased potential for long-term propagation, sustained viability through five passages—even in reduced medium—is insufficient as a criterion for successful malignant cultures.

One culture (PDTO39_EGF) showed a biphasic growth pattern, initially being classified as a low-MAF culture by NGS after initial rapid expansion of organoids until passage 3. Several pathogenic mutations were detected but all with MAF < 5%. At passage 4, most organoids in the culture declined rapidly except for a few with distinct morphology, which continued growth for the subsequent passages. Repeated NGS analysis was performed at passage 7, showing the same pathological mutations but this time with a high MAF of 50–100%.

This demonstrated that early sequencing of expanded organoids might inadvertently misclassify cultures.

### 3.4. CAFs Were of Myofibroblastic Phenotype

To characterize our CAF cultures, we performed flow cytometric analysis using the previously reported CAF markers alpha smooth muscle actin (αSMA), platelet-derived growth factor receptor alpha (PDGFRα), fibroblast-activated protein (FAP), and interleukin-6 (IL-6) [13,14,26]. The panel was chosen to determine the presence of CAF markers and distinguish between myofibroblastic (myCAFs) and inflammatory (iCAFs) phenotype as described by Ohlund et al. [26].

All cultures had a high fraction of both PDGFRα- and FAP-positive cells and none or only a negligent amount of IL-6-positive cells (Table 1). Four of six cultures also showed high αSMA expression, suggesting that the CAFs were of the myCAF phenotype, which was expected given their growth conditions on a plastic surface [26]. The remaining two cultures had fewer αSMA-positive cells. To exclude contamination with epithelial or tumor cells, one culture was further characterized by NGS analysis and IHC. No mutations or epithelial biomarkers were expressed, confirming fibroblast origin (Figure S2, Table S2). In the case of the last remaining culture, no additional material was available for supplementary analyses.

### 3.5. CAF/PDTO Co-Culture Model

**Figure 6:**
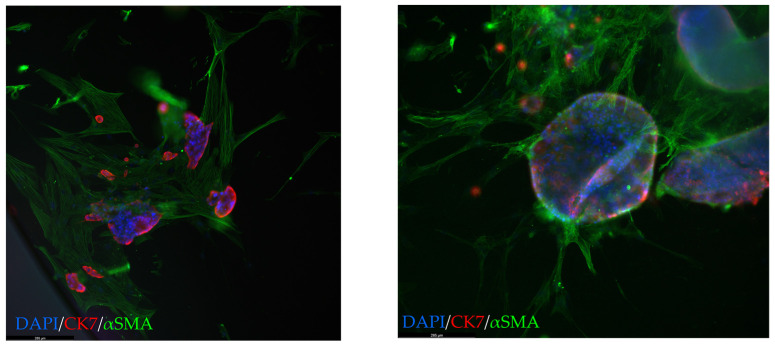
Co-culture (10× magnification). Green: αSMA; red: CK7; blue: DAPI. A close relationship between CAFs and PDTOs is seen. CAFs, cancer-associated fibroblasts; PDTO, patient-derived tumor organoid; CK7, cytokeratin 7; αSMA, alpha smooth muscle actin.

Co-cultures were grown in diluted (10%) Geltrex^®^ to avoid CAF inhibition by embedding them in BME, as previously shown [19,26], but still allowing PDTO/CAF contact and 3D culture formation of stromal/tumor cells. Patient-matched CAFs and organoids cultures could be generated in three cases; however, due to a global shortage of Geltrex^®^/Matrigel^®^ during the COVID pandemic, co-culture creation had to be postponed for all three. Once reagents were available again, either CAF or organoid cultures had become non-proliferative in two cases. When patient-matched CAFs were not available, the most recent CAF culture was used for co-culture creation. Co-cultures were established using both WTOs, namely low-MAF organoids and vPDTOs. Microscopic imaging and immunofluorescence revealed that CAFs formed a network around organoids (Figure 6), suggesting that CAF/PDTO could interact with each other in our model.

### 3.6. Isolating PDTO Viability in Mixed-Cell Culture

**Figure 7:**
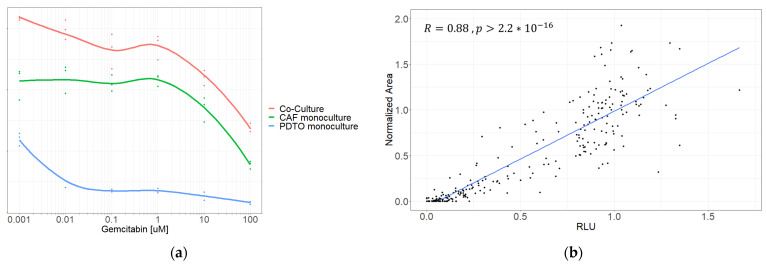
(**a**) Dose-response curve of co-culture and monocultures of CAFs and PDTOs, respectively, generated using CellTiter-Glo^®^ 3D. (**b**) Correlation between normalized area and luminescent signal.

For the system to function as a tool for drug screening in personalized medicine, we needed to be able to isolate PDTO from CAF viability in the co-culture. We performed initial drug screenings on several co-cultures along with corresponding monocultures of its constituents. We initially attempted to assess the PDTO response using CellTiter-Glo^®^ 3D (Figure 7a), but while comparing dose–response curves of co-cultures against the corresponding monocultures, it became evident that in cases where CAFs emitted a substantially higher signal than PDTOs, this would likely lead to misinterpretation of results.

Instead, the PDTO response was quantified using an image-metrics-based approach by manually measuring PDTO size on brightfield pictures, as previously reported [27,28,29]. To test whether this could accurately reflect the PDTO response, we compared viability obtained using image metrics against viability from CellTiter-Glo^®^ 3D in PDTO monocultures. The normalized viability obtained using image metrics correlated with relative luminescence (R = 0.88; *p* < 0.0001) (Figure 7b), validating the method as a classifier of viability.

### 3.7. CAF–Organoid Interaction

After establishing a method of isolating PDTO response, we investigated whether the addition of CAFs would impact the culture growth. Co-cultures were established and allowed to grow for 6 days in parallel with monocultures of its constituents, after which growth was evaluated using image metrics and CellTiter-Glo^®^ 3D.

**Figure 8:**
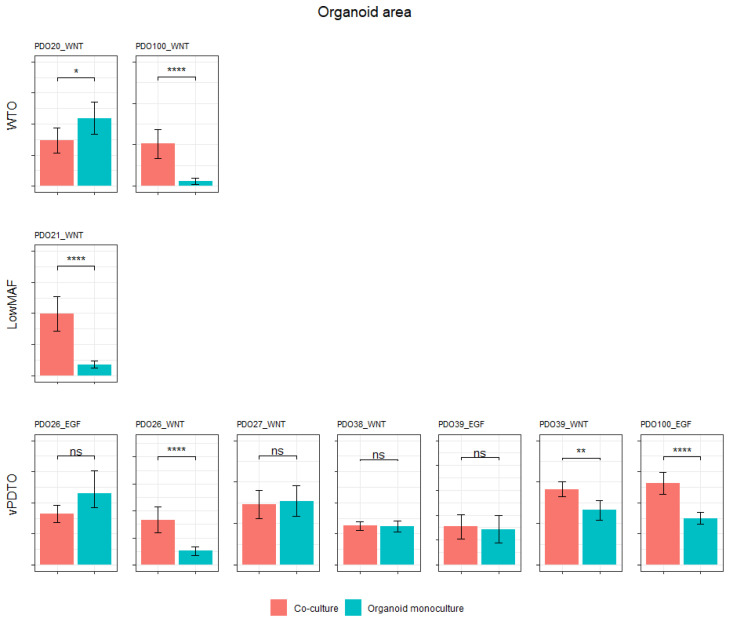
Mean area of organoids in co-culture vs. monoculture. Organoid area in co-culture vs. in monoculture. Graphs are scaled individually and do not share common *y*-axis. ns, not significant. * *p* ≤ 0.05, ** *p* ≤ 0.01, and **** *p* ≤ 0.0001.

In five out of ten co-cultures (three vPDTOs, one WTO, and one low-MAF culture), organoids grew significantly better compared to their monoculture counterparts as measured by area (Figure 8). This confirms that our model, at least in part, recapitulated CAF–PDTO interaction.

**Figure 9:**
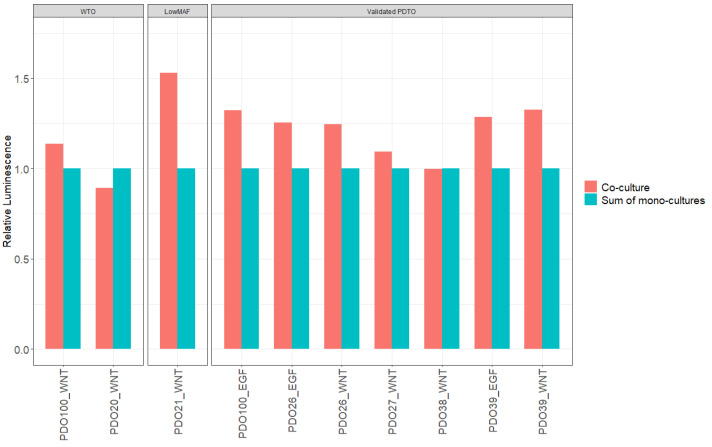
Luminescent signal obtained by CellTiter-Glo^®^ 3D. Comparison of co-cultures vs. sum of monocultures.

To further validate our results, we also examined if the CellTiter-Glo^®^ 3D signal obtained from the co-cultures could be described as the additive signal from monocultures of CAFs and PDTOs. The average luminescent signals from co-culture wells were compared to the sum of luminescence measured from corresponding monocultures (Figure 9). The mean luminescent signals from co-cultures were significantly larger than the additive signals from monocultures of PDTOs and CAFs in eight out of ten cases, with a mean difference of 21% (95% CI, 8.1–34.2 %; *p* = 0.005), further supporting that synergistic interactions on cellular growth did indeed occur in our co-culture model.

## 4. Discussion

We present our experiences with creating PDTO co-cultures from EUS-FNB samples for the potential use in personalized medicine. Although establishment of CAF and PDTOs from EUS-FNB samples from primary tumors is feasible, it is associated with significant challenges. While organoid establishment was observed in >90% of biopsies that were diagnostic for adenocarcinoma, and 50% of these could be expanded to yield enough biomass for downstream analysis (co-culture creation + drug screening + NGS analysis + histopathologic evaluation), only 19.2% had high purity of malignant cells (>30% MAF) and were long-term expandable in TSM. This success rate was significantly lower than described in several other publications [10,11,30]. Several reasons may explain this discrepancy. We included patients prior to confirmation of a histopathologic diagnosis of PDAC; therefore, tissue from several needle passes had to be used for primary diagnostics prior to obtaining tissue for co-culture establishment. This limited the number of needle passes for PDTO establishment due to safety and ethical reasons. Furthermore, a higher degree of blood contamination was observed following the diagnostic needle passes, which may have impacted cellularity assessment. Despite the introduction of FNB needles, which provide significantly more tissue compared to traditional FNA needles [31], the total tissue amount is still limited, impacting tumor cell yield for PDTO establishment. This is exacerbated in biopsies from highly desmoplastic tumors such as PDAC, where the stromal component may make up most of the tissue. Furthermore, establishing PDTOs from primary tumors is likely to increase the risk of contamination with benign cells compared to establishing PDTOs from distant metastases, where the host tissue has less organoid-forming potential. Interestingly, Grossman et al. [6] also attempted to establish PDTOs from primary PDAC using EUS-FNB samples from 27 patients, reporting a low success rate of PDTO expansion to drug screening (12.5%). Likewise, Beutel et al. [5] described an overall success rate of expanding PDTOs from PDAC to drug screening of 63%, but this was reduced to 27% when using percutaneous needle biopsies from primary tumors (despite using larger 16–18 G needles), suggesting that PDTO establishment from the primary lesions is associated with difficulties. Our CAF establishment rate was 25.6% from biopsies, which was acceptable, but the combined limited success rate for both techniques limited the patient-matched CAF and PDTO cultures to three.

One of our key findings was that thorough validation of PDTOs was necessary in order to confirm malignancy. Despite taking measures to limit inadvertent expansion of benign organoids by growing them in reduced TSM, this remained a significant problem, and 25% of the cultures we sent for downstream analysis came back negative for any pathologic mutations. Previous publications [7,8,10,32] have defined a successful PDTO as being viable through five passages, but in our experience this limit is insufficient to validate malignancy in biopsy-derived organoids from primary tumors, as several of our WTO cultures did remain viable past this limit. It has previously been shown that benign pancreatic organoids can be cultured for 20+ passages in complete organoid medium [23,24], so we expect this problem to be exacerbated when utilizing complete (non-reduced) medium. Others have defined a successful culture as generating sufficient material for drug screening experiments, but we showed that expansion alone—which was achieved for several cultures without malignant features—does not guarantee malignant characteristics. We also attempted to classify live organoids as malignant- or benign-based on morphology, but we observed a significant overlap between the two (Figure 4), rendering the method impractical. Histologic evaluation by trained pathologist had some value in determining malignancy, with high-grade nuclear and cellular pleomorphism only being observed in our vPDTO group. However, the morphological characteristics were not sensitive enough for identification of malignant cultures, as vPDTOs with low-grade pleomorphism were also noted (Figure 2). Of note, some histopathological characteristics usually associated with malignancy, such as increased nuclear-cytoplasmatic ratio and hyperchromatic nuclei, were present in all cultures, including one established from normal pancreatic tissue. Our pathologists scored most cultures to have moderate pleomorphism as well, including both normal reference and some WTO cultures. These findings suggest that simply growing cells as organoids introduces artefacts, which complicates histological evaluation.

Instead, we used NGS as a tool for classifying cultures. We used a custom panel to detect hotspot mutations in 51 selected genes. While comprehensive sequencing of all genes was not performed, leading to a theoretical possibility of misclassification, we did observe that all cultures classified as malignant (vPDTOs) grew markedly better without signs of reduced proliferation during later passages. This led us to conclude that our classification system was correct and sufficient.

Isolating PDTO viability in co-cultures provides obvious challenges when using standard assays such as CellTiter-Glo^®^ 3D. To circumvent this, we used PDTO area on brightfield pictures as a proxy for organoid health. Attempts to extract these data using automated methods such as OrganoSEG (https://github.com/JanesLab/OrganoSeg) (Accessed on June–July 2022), IncuCyte, (v2020c) and Cellprofiler v4.2.1 software were unsatisfactory, failing to distinguish between aggregates of dead and viable cells or between CAFs and PDTOs. Instead, PDTOs were manually identified in CellProfiler software, which was laborious and time-consuming but yielded acceptable results. After reviewer training, we validated the results by comparing the normalized area with the normalized CellTiter-Glo^®^ 3D signal in monocultures of PDTOs and observed high correlation. The advantages of the technique are that it can be readily performed and also retrospectively on simple bright-field microscopic images. The main limitation of the method is that it is time-consuming, preventing high-throughput screenings and is therefore not practical for clinical applications. For the method to be implementable in a clinical sense, automated methods such as AI classification would need to be applied. Schuth et al. [19] recently published results on the drug screening of co-cultures using an elegant setup of fluorescent cell trackers, DAPI, and propidium iodide measured on a confocal microscope. While this method requires access to advanced confocal microscopy, this setup does appear as an appealing alternative to manual measurements of PDTO viability.

Our co-cultures were grown in diluted Geltrex^®^ for two purposes: it allowed for direct drug screening, as previously described by Tiriac et al. [7], lowering the Geltrex^®^ cost. Secondly, it circumvented issues with induction of quiescence in CAFs when seeded in undiluted Geltrex^®^. We observed direct contact between CAFs and PDTOs, and we showed that in 50% of the cases, CAF–organoid interaction positively affected organoid growth across all groups in our model. We also investigated our cultures with CellTiter-Glo^®^ 3D measurements and observed a larger luminescence reading from co-cultures than the sum of luminescence from corresponding CAF and PDTO monocultures. While it is impossible to determine whether the difference stems from increased growth of CAFs or organoids, it is suggestive of a synergistic interaction between the two cell types in the present co-culture model.

This study has several limitations. Firstly, in five (50%) of our co-cultures, the addition of CAFs did not affect viability of PDTOs as measured by area despite the CellTiter-Glo^®^ 3D signal being higher in the co-culture than the combined signal from monocultures. This could indicate that viability measurements using image metrics are not as sensitive as standard commercial assays and that one should exercise caution when interpreting the results in a co-culture setting. Another reason for the variance in PDTO/CAF interaction might be due to CAF heterogeneity. As mentioned, whenever patient-matched CAFs were unavailable, the most recently established CAF line was used for creation of co-cultures. While this did introduce a certain heterogeneity between experiments, we elected to use primary CAFs over an immortalized cell line, as some differences in phenotype have been demonstrated [33] between primary cultures an immortalized cell lines. As each CAF line was only used in a few co-culture experiments, the study was underpowered to determine any correlation between CAF characteristics (Table 1) and strength of interaction between CAFs and PDTOs.

Another explanation for the variance in interaction might be due to tumor subtypes, as defined by Moffit et al. [34]. Whereas Shinkawa et al. [35] showed that highly differentiated tumors of the classical subtype showed growth dependency on niche factors secreted by CAFs, tumors of a more basal-like subtype did not, providing a possible explanation for the variance observed.

Lasty, we did not focus on the impact of tumor medium in this study. The use of reduced TSM-WNT^+^ and TSM-EGF^+^ was to limit expansion of normal epithelium but may impact CAF–PDTO interaction differently. While we did not observe any significant difference in our different media in their ability to affect PDTO growth in co-culture conditions, it should be noted that media components such as TGF-β inhibitor A83-01, which was a constituent of TSM-WNT^+^ only, could play a significant role in CAF phenotype, as demonstrated by Harryvan et al. [36], and possibly affect interaction with PDTOs. Further research is warranted to uncover the optimal media for studying CAF–PDTO interaction.

## 5. Conclusions

In conclusion, we showed that both PDTO and CAF cultures can be established from EUS-FNB samples of PDAC, with the possibility of creating patient-matched models. We created a co-culture setup that allows for interaction between PDTOs and CAFs, improving the viability of PDTOs. The co-culture setup readily allows for drug screening of multiple compounds utilizing simple image metrics with reasonable precision to isolate the PDTO response in a mixed human cell culture.

This model allows for potential comparison between co-culture response and clinical outcome in treatment-naïve patients with pancreatic cancer, but further research is warranted to elucidate whether it provides better predictions over standard PDTO models.

Furthermore, we demonstrated that standard validation techniques such as prolonged viability for 5+ passages, histologic evaluation, or cellular expansion each on their own are insufficient to guarantee the malignancy of PDTO cultures established from EUS-FNBs from primary tumors.

## Figures and Tables

**Table 1:** Expression of CAF markers in different CAF cultures. Numbers are given as percentage of positively stained. Cut-off is calculated as fluorescent intensity higher than the 99th percentile of isotype controls.

	IL-6 (%)	αSMA (%)	PDGFRα (%)	FAP (%)
PDO4-CAF	0.30	74.20	86.30	60
PDO23-CAF	0.18	86.70	74.80	97.50
PDO29-CAF	0	66.90	26.50	94.90
PDO31-CAF	0	10.30	79.10	98.10
PDO34-CAF	0	11	85.50	98.30

## Data Availability

The data presented in this study are available in this article (and supplementary material).

## Supplementary Materials

The following supporting information can be downloaded at: https://www.mdpi.com/article/10.3390/cancers15143677/s1. Section S1: Supplementary Materials and Methods. Figure S1: CNV analysis of PDO20_WNT showing no sign of chromosomal instability. Figure S2: IHC stain of CAF culture PDO34-CAF against fibroblast marker vimentin. Table S1: NGS data of cultures PDO_20 – PDO_100. Table S2: NGS data for CAF culture PDO34_CAF. File S1. Raw Data. References [37,38,39] are cited in the supplementary materials.
