# Supplementary material for: Validation of a Novel EUS-FNB-Derived Organoid Co-Culture System for Drug Screening in Patients with Pancreatic Cancer
Source: Cancers (Basel). 2023 Jul 19;15(14):3677. doi: 10.3390/cancers15143677 (PMC10377599; doi:10.3390/cancers15143677)
Supplement: Supplementary file 1 [file cancers-15-03677-s001.zip › cancers-2459003-supplementary.docx]

Supplementary Materials

Section S1. Supplementary Materials and Methods

Section S1.1. Drug Screening Experiments

Drug screening experiments were performed on co-cultures as well as monocultures of PDTOs and CAFs. Cultures were allowed to recover for 1 day after plating on 384-well plates, after which serial dilutions of chemotherapeutical agents were added in concentrations ranging from 10^−9^ to 10^−4^ M in 10-fold increments using the Echo 525 acoustic liquid handler (Beckman Coulter Life Sciences, Indianapolis, IN, USA). Each condition was tested in triplicates. Choice of chemotherapeutical agents and combinations were based on treatment regiments commonly used for pancreatic cancer (Gemcitabine, Nab-Paclitaxel, Irinotecan, 5-Fluorouracil (5-FU), and Oxaliplatin, including combinations Gemcitabine/Nab-Paclitaxel and 5-FU/Irinotecan/Oxaliplatin). Images of wells were acquired on day 0 to assess irregularities in plating density and after 5 days of drug incubation (day 6). On day 6, the viability of co-cultures was assessed using IncuCyte^®^ imaging and CellTiter-Glo^®^ 3D.

Section S1.2. Immunofluorescent Staining

Co-cultures were stained using immunofluorescence. PDTOs were grown with CAFs in ratio 2:1 in TSM with 10% Geltrex^®^ for 10 days in 96-well plates. The medium was removed, and cells were fixated using formaldehyde 4% for 30 min. Cells were permeabilized using 0.25% Triton X-100 ([9036-19-5](https://www.sigmaaldrich.com/DK/en/search/9036-19-5?focus=products&page=1&perpage=30&sort=relevance&term=9036-19-5&type=cas_number), Sigma-Aldrich) for 10 min, and blocking solution of 3% bovine serum albumin in PBS was applied for 1 h at room temperature. Cells were incubated with mouse mAb to smooth muscle actin alpha (αSMA) (ab7817, Abcam) 1µg/mL and rabbit mAb to CK7 (MA5-33049, ThermoFisher) 1:500 diluted in blocking solution overnight at 5 °C. Following washing, co-cultures were incubated with Goat anti-Mouse IgG, IgM AlexaFluor 488 (A-10680, ThermoFisher) 1:1000, and Goat anti-Rabbit IgG Alexa Fluor 568 (A-11011, ThermoFisher) for 1 h. Intermediate washing with PBS was gentle to prevent loss of cells attached to the bottom of the wells.

Section S1.3. Immunohistochemistry and Assessment of Morphology

PDTOs were collected from wells by aspiration of Geltrex^®^ domes in 1000P pipettes together with tumor medium. The suspension was washed with AdDF+++ and centrifuged at 800 rpm and the supernatant removed. The resulting cell pellet was resuspended in 50–100 µL Epredia™ Histogel™ (22-110-678, ThermoFisher), which had been liquified by heating to approximately 60 °C and allowed to cool for 2–3 min. The resulting cell pellet/Histogel suspension was transferred to a cassette and allowed to solidify at 5 °C for 30 min before transferring to 4% formaldehyde; then, it was left to fixate overnight prior to embedding in paraffin. The formalin-fixed paraffin-embedded (FFPE) tissue blocks were sectioned with a microtome and initially stained with hematoxylin and eosin (HE). Subsequently, immunohistochemical staining was performed with antibodies against IPM3, Maspin, s100p, and Ki67, based upon Jensen et al*.* [37], as well as p53 and SMAD4 using an automated system (Ventana Benchmark Ultra with UltraView/Optiview detection kit (760-500/760-700), Roche, Basel, Schweiz, or Omnis with EnVision Flex+ detection kit (GV800), Agilent, Glostrup, Denmark) according to the manufacturer’s instructions. The slides were digitized using a NanoZoomer XR (Hamamatsu, Hamamatsu City, Shizuoka Pref., Japan) slide scanner and NDP-viewer software (Hamamatsu) and reviewed by trained pathologists regarding morphology and cytoarchitecture.

Section S1.4. CAF Phenotyping by Fluorescence Activated Cell Sorting (FACS)

A total amount of 2 × 10^5^ CAFs per sample were analyzed by FACS. Firstly, cells were detached by trypsinization as described above, washed, and incubated for 10 min at room temperature with Fc-blocking reagent (422301 Human TruStain FcX, Biolegend, San Diego, CA, USA) diluted 1:10 in FACS buffer. Next, cells were washed and stained with extracellular antibodies FAP-PE, PDGFRa-BV421 (both 5 µL/sample), and dead cell exclusion dye, either LIVE/DEAD™ Fixable Near-IR Dead Cell Stain Kit (L10119, ThermoFisher) or Fixable Viability Stain 510 (564406, BD Biosciences, Franklin Lakes, NJ, USA). Cells were stained at 4 °C in the dark for 30 min, washed twice in FACS buffer, and then fixated and permeabilized for 30 min using 200 µL eBioscience™ Fixation/Permeabilization reagent (88-8824-00, ThermoFisher). Next, cells were washed twice in fixation-permeabilization buffer and then stained with αSMA-APC and IL6-FITC antibodies (both 5 µL/sample) for 30 min in dark. Cells were again washed ×2 and re-suspended in fixation-permeabilization buffer before acquisition on a Novocyte Quanteon flow cytometer (Agilent), with a relevant compensation control applied. Isotype controls were used for both BV421, PE, APC, and FITC conjugated antibodies in some experiments but were later skipped due to the absence of nonspecific binding of isotype control antibodies.

Section S1.5. DNA Purification and Next-Generation Sequencing

DNA was isolated from a cell suspension containing the organoids using an in-house raw extraction method (Proteinase K and Tris EDTA) and quantified using the Qubit 2.0 fluorometer High-sensitivity kit (Q32854, ThermoFisher). A total of 51 cancer-related oncogene and tumor suppressor genes were investigated on the material using the Ion AmpliSeq Cancer Hotspot Panel version 2 (4475346, ThermoFisher), which covers the most frequent hotspot mutations in the following 50 genes: ABL1, AKT1, ALK, APC, ATM, BRAF, CDH1, CDKN2A, CSF1R, CTNNB1, EGFR, ERBB2, ERBB4, EZH2, FBXW7, FGFR1, FGFR2, FGFR3, FLT3, GNA11, GNAQ, GNAS, HNF1A, HRAS, IDH1, IDH2, JAK2, JAK3, KDR, KIT, KRAS, MET, MLH1, MPL, NOTCH1, NPM1, NRAS, PDGFRA, PIK3CA, PTEN, PTPN11, RB1, RET, SMAD4, SMARCB1, SMO, SRC, STK11, TP53, and VHL. Additional regions in the genes BRAF, CDKN2A, KRAS, RNF43, TP53, SMAD4, and GNAS were added to the panels (as described in previous publications [38,39]*)*. Targeted NGS was conducted on the S5+ System (ThermoFisher) with the custom-made panel and according to the manufacturer’s instructions. Library preparation was performed from 10 ng of purified DNA using Ion AmpliSeq Library kit 2.0 (4480441ThermoFisher) according to the manufacturer’s instructions. The Ion 510/520/530 kit—Chef (A34461, ThermoFisher) was used as template kit for the IonChef (ThermoFisher). Sequencing was carried out using the Ion S5 sequencing kit with an Ion 530 Chip (ThermoFisher). Sequenced data were initially aligned and mapped to the human hg19 reference genome using the Torrent Suite Server (v5.14) (ThermoFisher) with default parameters. The Ion Reporter Workflow (v.5.14) (ThermoFisher) was used to perform variant calling of the DNA libraries. High-confidence somatic variants were visually confirmed in the Integrative Genomics Viewer version 2.5.2 (Broad Institute of MIT and Harvard). Manual inspection of KRAS and TP53 genes was performed to check for mutations with MAF < 5%.

Section S2. Supplementary Figures


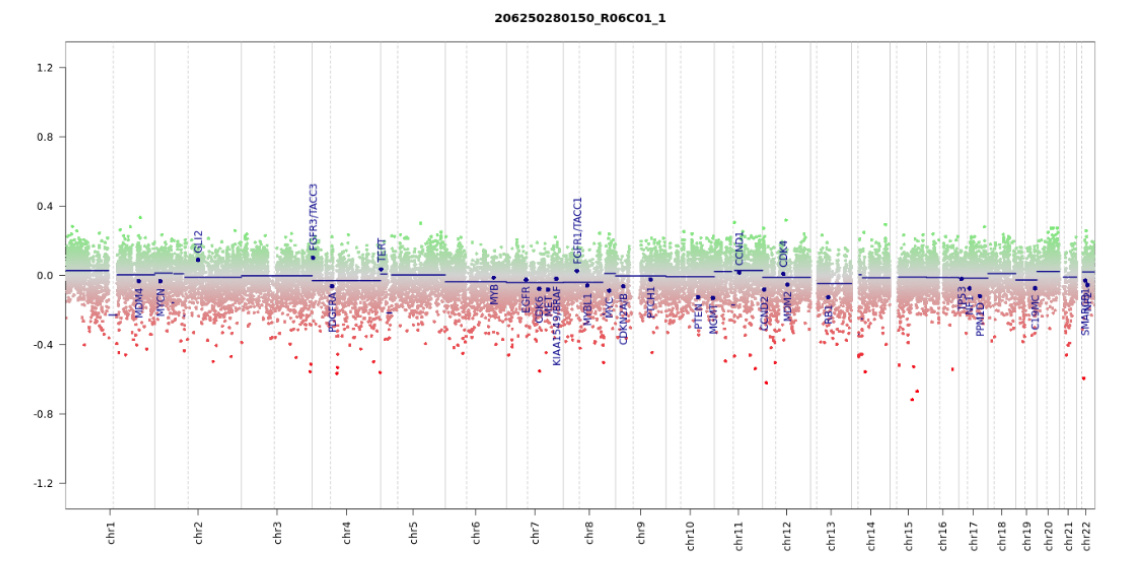


**Figure S1.** CNV analysis of PDO20_WNT showing no sign of chromosomal instability.


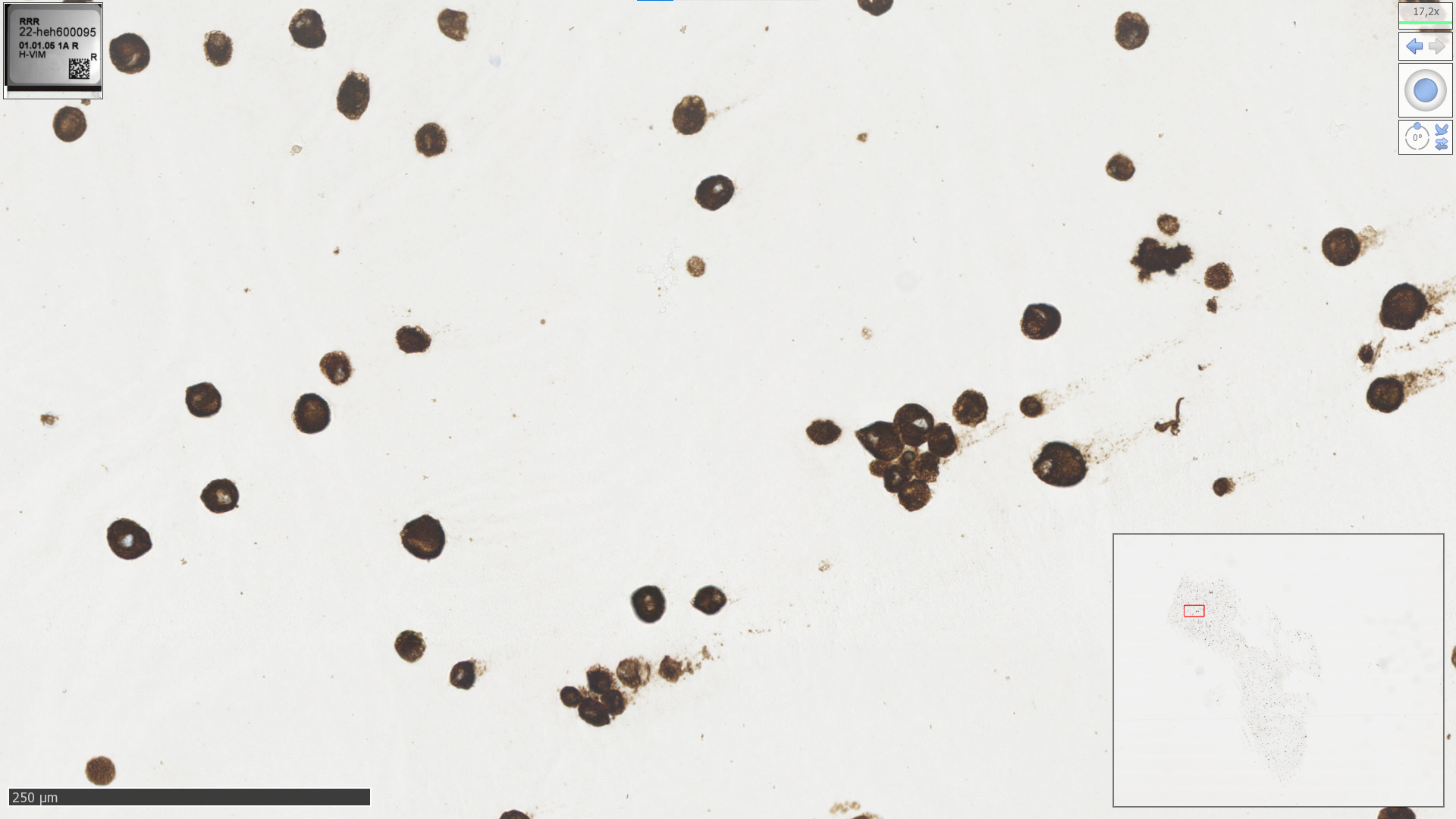


**Figure S2.** IHC stain of CAF culture PDO34-CAF against fibroblast marker vimentin.
